# Potential negative effects of the installation of video surveillance cameras in raptors’ nests

**DOI:** 10.1038/s41598-022-26153-7

**Published:** 2022-12-20

**Authors:** Pascual López-López

**Affiliations:** Movement Ecology Lab, Cavanilles Institute of Biodiversity and Evolutionary Biology, University of Valencia, C/Catedrático José Beltrán 2, 46980 Paterna, Valencia Spain

**Keywords:** Behavioural ecology, Biodiversity, Conservation biology, Animal behaviour

## Abstract

Video surveillance cameras installed on birds’ nests are a cost-effective tool to study many aspects of ecology and behaviour that would otherwise be practically impossible to obtain. However, although most studies report neutral effects of cameras on birds, very few studies analyse in detail the potential negative effects of their use, particularly on raptors. Here, using a long-term database of a population of Bonelli’s eagle (*Aquila fasciata*) collected from 2000 to 2022, I show how the inappropriate use of video surveillance cameras could result in negative effects on the reproduction of a threatened species through a before-and-after control-impact study design. Pairs under video surveillance showed lower productivity, lower breeding success and unusual delayed laying dates. The installation of cameras close to the laying date, coinciding with the mating phase of individuals, most of them subadult inexperienced birds; in combination to the reiteration of visits to the nests once the cameras were installed to check the system, particularly during the incubation period and early stages of breeding; and the installation of cameras in a particular area subject to constant human disturbance, might explain these results. Potential management actions to mitigate the effect of the installation of video cameras on birds’ behaviour should include the need to plan the intervention dates, testing the systems beforehand under controlled conditions and adequate post-installation monitoring to avoid unnecessary disturbance to animals. Finally, I urge the scientific community to report the potential negative effects observed in their studies, especially if the target species are threatened with extinction.

## Introduction

The use of video surveillance cameras installed on nests is a cost-effective tool to study many aspects of the breeding ecology and behaviour of birds^1^. The use of camera traps, trail cameras, video cameras, and other remote monitoring devices has made it possible to investigate many aspects of the reproductive ecology of birds that would otherwise be difficult to address through direct observation^2^. In particular, the use of remote cameras installed in nests allows for getting detailed information on animal behaviour including continuous monitoring throughout the breeding season^3–5^, nest predator identification^6–8^, prey identification^4,9–13^, behavioural studies of nesting birds^14–19^; and even to promote public participation in citizen science projects to study birds’ diet^20^.

Remote monitoring of nests using electronic devices is a technique with decades of experimentation. The first studies date back to the fifties of the last century^21,22^ and technological innovations have allowed significant advances in improving both the quantity and the quality of the images received, going from the first prototypes based on 35 mm photographic cameras to video surveillance cameras remotely controlled that allows real-time monitoring and public access through the Internet (review in^23^). Although most studies report neutral effects of the use of cameras inside nests (e.g.,^5,18,24–27^), very few studies report the possible potential effects that the installation of electronic devices inside nests might cause on behaviour (e.g. avoidance, decrease in the frequency of visits to the nest, nest abandonment) or direct and indirect effects on breeding performance (e.g. increase or decrease in predation risk, reduction in productivity of remotely-monitored nests). For example, among the few studies that exist about this topic, some have reported evidence that the use of camera equipment may reduce nest predation rates^28^, might bias data collected on predator identity (review in^29^), or even could cause nest abandonment in extreme cases^30^. Other studies, in turn, show that, in general, the cameras provide interesting valuable results but alter the behaviour of breeders, at least temporarily^13^, whereas others report shorter habituation time in those birds with more experience in front of the cameras^31^. Ultimately, several studies report technological limitations and usual technical failures that make it necessary to check the cameras continuously, with the potential risk of disturbance at nests^31–33^.

At the end of 2020, upon the request of some forest rangers, the regional government of the Valencian Community (Generalitat Valenciana, Spain) began a project consisting of the installation of video surveillance cameras in Bonelli’s Eagle (*Aquila fasciata*) nests in the south of the Castellon province (eastern Spain) to study eagles’ diet. This species is currently highly threatened in Spain, being the only species of a large bird of prey that is experiencing an effective regression of its population in many parts of the country^34^. Specifically, at the regional level, the species has gone from being considered a “vulnerable” species to “endangered of extinction” in the Valencian Community^35^. This change in risk category was based on a population viability analysis in which, fundamentally thanks to the combined use of GPS transmitters and long-term field monitoring, it was found that the population trend is compromised in the medium and long term due to a high pre-adult and adult mortality, analogous to that reported in other neighbouring geographic regions^36–38^. In particular, since 2000, the Bonelli’s eagle population has suffered a decline of 48.6% in the region, with an observed reduction of the breeding population from 31 territories occupied in 2000 to 18 occupied in 2022^39,40^. Specifically, in the south of this geographical region, video surveillance cameras were installed by the regional government in the latest two breeding seasons, in an area of high adult mortality^41^ and where the recreational use of the environment for leisure activities has increased significantly in recent years, which has resulted in a significant increase in disturbance to wildlife in general and the endangered Bonelli’s eagle in particular^42^.

Since the year 2000, I have been collecting an annual monitoring database of the Bonelli’s eagle population in the study area in which information on occupation, breeding performance and demographic parameters is systematically collected thanks to the combination of field monitoring techniques, bird ringing and telemetry information^39,41–43^. Taking advantage of this long-term database, the objective of this work is to analyse the variation in the reproductive parameters of the Bonelli’s eagle through a before-and-after control-impact (BACI) methodology^44,45^ in territories in which video surveillance cameras were installed and in control territories monitored by field observation throughout the 23-years study period. Once reported the results of this work, it is intended to inform the regional government about the possible causes that could be behind the decrease in breeding performance observed in pairs in which video surveillance cameras were installed. Furthermore, I discuss the advantages and potential drawbacks of the use of video-surveillance cameras to study raptors in a general context.

## Methods

### Study area

The study area is located in eastern Spain and includes the entire Castellón province. The area covers 6612 km^2^ and ranges in elevation from the sea level to 1814 m of the highest mountain. The climate is Mediterranean, with annual mean temperatures varying from 17 °C along the coast to 8 °C in the inner highlands. The dominant landscape is composed of Mediterranean scrublands dominated by *Pistacia lentiscus, Rosmarinus officinalis, Chamaerops humilis*, among others, oak forests (*Quercus faginea, Quercus suber*) and Mediterranean evergreen forests (*Quercus ilex, Pinus halepensis, Pinus nigra* and *Pinus sylvestris*). Further details on the description of the study area are available at^39,46,47^.

### Field information and breeding parameters

I have been monitoring Bonelli’s eagle population every year from 2000 to 2022. During each breeding season, all known and potential territories based on suitable habitat for the species^47^ were visited at least three times: one at the start of the breeding season in January–February to check occupancy, courtship behaviour and egg-laying; a second one at the middle of the breeding season in March–April to check early breeding failure, confirm the onset of reproduction and record the first chicks; and a third visit at the end of the breeding season in May–June to confirm breeding success and nestlings’ age based on feather development^48^. All observations were made 300 m away from cliff nest sites with 20–60× field spotting scopes (Leica Televid 77 and Swarowski AT80 telescope) during clear days to avoid disturbance to eagles. A territory was considered to be occupied if it was observed nests with green branches, typical pair behaviour, courtship, brood rearing activity or nestlings^39^. Pair composition was also recorded. To this end, the age of the breeding individuals was recorded according to plumage characteristics. Birds were considered subadults if they were in their third and fourth calendar-year and adults if they were from the fifth calendar-year onwards^49^. Juveniles in their first and second calendar-year do not breed since sexual maturity is usually reached in their third or fourth calendar-year. Once recorded field data, breeding information was summarized into the following reproductive parameters as usually reported in raptors’ literature: productivity = fledged chicks/pair; breeding success = successful pairs/pairs initiating reproduction (i.e., pairs that laid at least one egg or exhibit incubating behaviour) (further details in^39^). Chicks were considered as fledged once reached the 80% of the normal development (i.e., when they were older than 55 days old taking into account that the first flight usually takes place when chicks are between 65 and 70 days old).

### Camera installation

**Figure 1:**
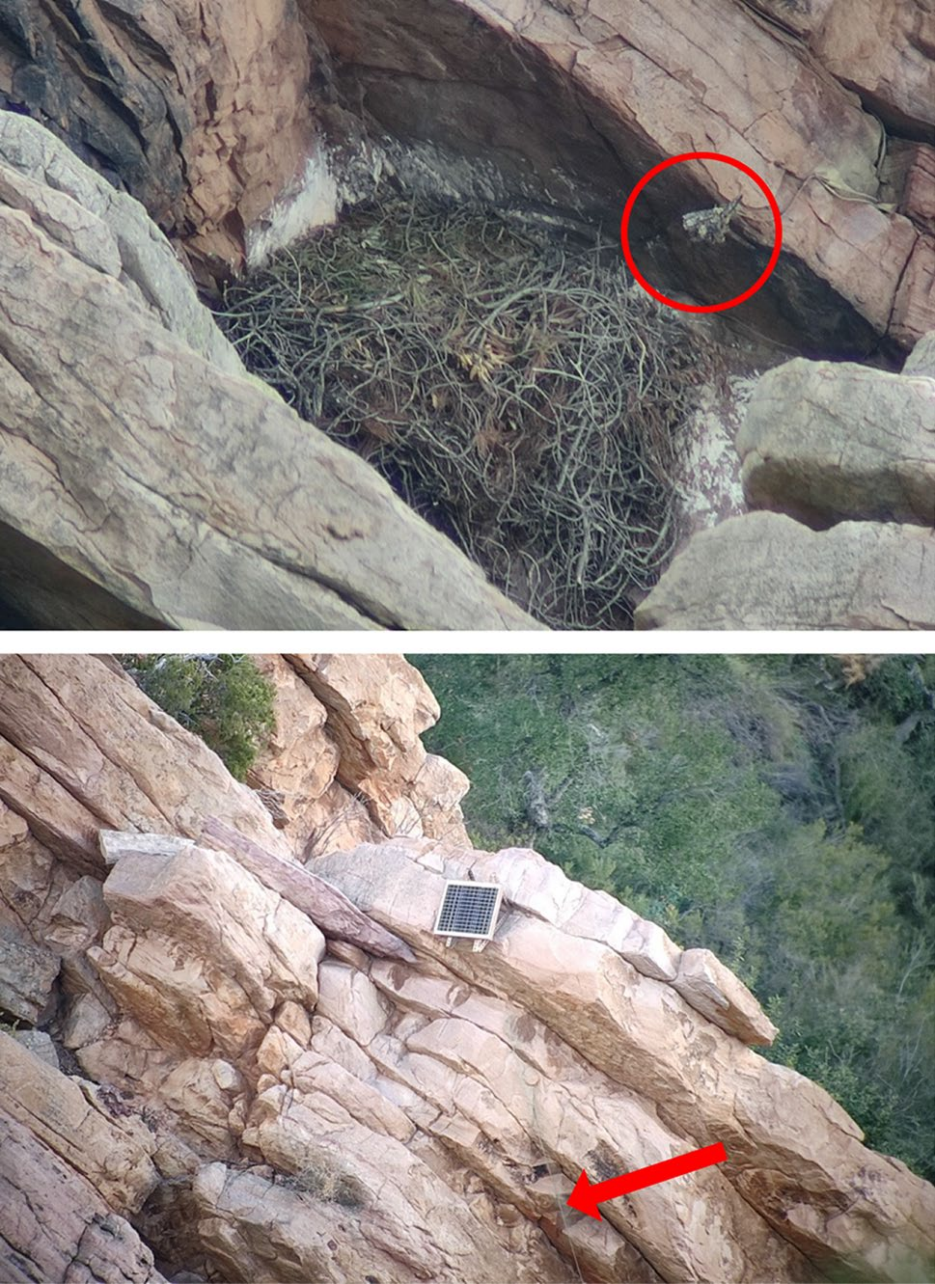
Video surveillance camera (circled in red) installed in a Bonelli’s eagle nest in eastern Spain (top panel). Solar panel and wiring (red arrow) installed just in the vertical of the same nest about 15 m above (bottom panel). In this case, the nest was arranged with green branches although the pair eventually failed reproduction. Pictures taken through field spotting scope on 4th April 2022. Credit: Pascual López.

Video surveillance cameras were installed by regional forest rangers in Bonelli’s eagle’s nests located in the “Sierra de Espadán” Natural Park and its surroundings (southern Castellón province) (Table 1). According to the combination of my field observations and the information requested to the regional environmental authorities in charge of the monitoring project (Generalitat Valenciana), four cameras were installed in late 2020 in four different territories and four cameras were installed in late 2021 in four additional territories. Detailed information on installation dates, operating years and specific issues reported by forest rangers of each intervention is reported in the Table 1. According to the information facilitated by the environmental authorities, four cameras were operating all over the 2021 breeding season and eight cameras during the 2022 breeding season (Table 1). The cameras were installed inside the nests at a distance of between 1 and 3 m from the centre of the nest. In most cases, a single camera was installed in one nest within each of the monitored territories per year. Nonetheless, in two cases, cameras were initially installed in one nest and were then removed and installed afterwards in a different nest once checked that the pair was arranging a different nest within the same territory. The cameras were powered by a solar panel connected to a battery or, in cases where there was no direct exposure to the sunlight, by a battery directly (Fig. 1; a detailed diagram of the video-monitoring system is available in Fig. S1, Supplementary Materials). The cameras sent the information to a device that sent the signal through the mobile telephone network and allows users to observe the images live. The solar panels, the batteries and the data transmission equipment were installed in the upper part of the cliff, just vertical to the nest (Fig. 1). All the nests were located on cliffs and to access nests it was necessary to descend into them by members of the work at height group of the regional forest rangers. Unfortunately, the specific brand as well as the technical details of the cameras, including the external dimensions of the whole camera set, the diameter of the objective or the type of illumination (e.g., infra-red wavelength) were not provided by the regional authorities despite the specific request made by the author of this study.

**Table 1:** Summary information of the video-surveillance cameras installed to monitor Bonelli’s eagle reproduction in eastern Spain in 2021 and 2022.

Pair	Installation date	Operation year	2021		2022	
			Fledged chicks	Observations	Fledged chicks	Observations
1	11/11/20	2021–2022	1	Unusual delayed laying date (14/03/21)	0	Pair members arrange the nest. No egg laying. Failure of the SIM card that needed reparation after camera installation
2	16/11/20 18/11/21	2021–2022	2	Unusual delayed laying date (17/03/21)	0	The two chicks die at the age of 3–4 days after a period of heavy rain. Power cable failure that needed reparation after camera installation
3	19/11/20 15/11/21	2021–2022	0	Pair members arrange the nest. No egg laying	0	Pair members arrange the nest. No egg laying. No technical issues
4	28/10/20 03/11/20 12/11/21	2021–2022	0	No egg laying. The pair changed the nest and moved to another cliff located 4.35 km from the breeding site occupied in the last two decades	2	Successful reproduction in the new breeding site. Lack of phone signal
5	29/11/21	2022	2	No camera installed	2	Successful reproduction. No technical issues
6	19/11/21	2022	1	No camera installed	0	The only chick dies at the age of 16 days after a period of heavy rain. Lack of phone signal
7	16/11/21	2022	2	No camera installed	0	Pair members arrange the nest. No egg laying. The antennas were broken by Iberian ibex (*Capra pyrenaica*) and needed reparation after camera installation
8	17/11/21	2022	0	No camera installed	0	No egg laying. Lack of phone signal. Insufficient solar power supply that required the installation of a power battery. Eurasian Griffon Vultures (*Gyps fulvus*) usurp the nest after lack of egg laying of the pair

### Statistical analysis

Descriptive statistics were computed as mean and standard deviation for all variables. Differences in productivity and breeding success before and after the installation of video surveillance cameras were tested through non-parametric Wilcoxon rank-sum tests with continuity correction. Generalized Linear Mixed Models (GLMM)^50^ were used to analyse the variation in productivity in relation to the installation of video surveillance cameras (“camera”), pair composition (“age”) and their interaction. Considering the nature of the dependent variable (i.e., count data ranging from zero chicks to two), the productivity was modelled using a Poisson distribution and log link. The variables “territory” and “year” were included as random terms to account for the non-independence of data^51^. Five models were fitted: (i) a null model fitted only with random terms; (ii) and (iii) two models including the single effect of each predictor variable (“age” and “camera”), separately; (iv) a model including their additive effect (“age” + “camera”); and (v) a model including the combination of the additive effects and the potential multiplicative effects of the combination of variables of interest (e.g. “camera” * “age”). All models were computed using the package “glmmTMB” for R^52^. The GLMMs were compared and ranked through an information-theoretical approach based on Akaike’s Information Criterion (AIC). The conditional and marginal R^2^ of all GLMMs were calculated using the lognormal approximation following^53^ in order to assess the relative contribution of random and fixed effects through the R package “MuMin”^54^. All computations and figures were done in R version 4.1.2. (https://www.r-project.org) with the “ggplot2” package. Statistical significance was set at α < 0.05.

### Ethics declaration

All field methods were carried out in accordance with relevant guidelines and local regulations to avoid disturbance to birds. This is an observational study. Therefore, there is no need for the ethical approval of any licensing committee.

## Results

Overall, 496 breeding attempts were recorded in 33 different territories in which 515 chicks fledged during the period 2000–2022. Population size decreased significantly from 31 occupied territories at the start of the study period to 18 occupied territories at the end (linear regression: F_1,21_ = 358.7, p < 0.0001, R^2^ = 0.945).

Two of the four pairs under video surveillance failed reproduction in 2021 and six of the eight pairs under video surveillance failed in 2022. One of these six pairs raised two chicks that died at the age of three and four days and other pair raised one chick that died at the age of 16 days after a short period of heavy rain. Most unsuccessful pairs failed before egg laying, although most of them arranged their nest with green branches (detailed information in Table 1).

The two pairs under video surveillance that bred successfully in 2021 showed unusually delayed laying dates (14th March and 17th March) in comparison to the average laying date of the overall population (18th February ± 13 days; period: 2000–2022; N = 199). Furthermore, these laying dates were also delayed in comparison to the average laying dates of these particular territories all over the study period (21th February ± 16 days and 15th February ± 12 days, respectively). On the other hand, the two pairs under video surveillance that bred successfully in 2022 showed laying dates one within the average values of the population (7th February) and the other one unusually advanced (22th January) in comparison with the average values of the population. Nonetheless, these values were within the average values of the same territories all over the study period (21th February ± 17 days and 11th February ± 12 days, respectively).

**Figure 2:**
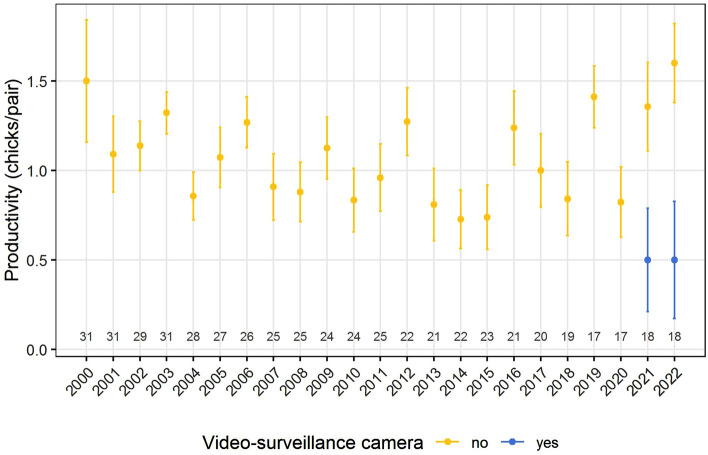
Annual variation in Bonelli’s eagle average productivity measured as fledged chicks per nest in the Castellón province (eastern Spain) from 2000 to 2022. Error bars show standard error. The number of pairs per year is shown at the bottom of the plot. Those nests under video surveillance (blue) showed the lowest values of productivity across the long-term temporal dataset. The productivity values are descriptive statistical values, not estimates yielded by any model.

Average productivity of the whole population was 1.04 ± 0.84 chicks/pair (n = 496; period 2000–2022). Considering all territories, overall average productivity did not vary in the study area before (1.03 ± 0.84 chicks/pair, n = 460; period: 2000–2020) and after (1.14 ± 0.93 chicks/pair, n = 36; period: 2021–2022) the installation of video surveillance cameras (Wilcoxon test: W = 7653, p = 0.422). Considering the two breeding seasons in which surveillance cameras were operating (2021 and 2022), there were no general differences in productivity between years (2021: 1.17 ± 0.92 chicks/pair, n = 18; 2022: 1.11 ± 0.96 chicks/pair, n = 18; W = 166.5, p = 0.889). However, in the same period, there were significant differences in the productivity between territories under video surveillance (2021: 0.50 ± 0.58 chicks/pair, n = 4; 2022: 0.50 ± 0.93 chicks/pair, n = 8) and those without video surveillance (2021: 1.36 ± 0.93 chicks/pair, n = 14; 2022: 1.60 ± 0.70 chicks/pair, n = 10) (W = 223, p = 0.002) (Fig. 2).

Considering the whole dataset, the results of the multi-model comparison of the GLMM showed that the two top-ranked models (i.e., those with ΔAICc values within two units) included the age and camera as predictors. The two top-ranked models showed one differences in productivity among territories in relation to pair composition (i.e., lower productivity in nests occupied by two subadults or by one adult and one subadult in comparison to those occupied by two adults); and the other one the additive effect of pair composition and the installation of video surveillance cameras (i.e., lower productivity in video-surveilled nests) (Table 2). Fixed effects of the two top-ranked models explained 87.19% and 87.78% of the overall variance (R^2^_marginal_ = 0.8719 and R^2^_marginal_ = 0.8778), respectively; and 87.70% and 88.23% of the variance was explained by the entire model, including both fixed and random effects (R^2^_conditional_ = 0.8770 and R^2^_conditional_ = 0.8823), respectively. Remarkably, those nests under video surveillance showed the lowest values of productivity across the long-term (23-years) temporal dataset (Fig. 2).

**Table 2:** Model comparison results of the Generalized Linear Mixed Models (GLMMs) of the productivity (measured as the number of chicks per nest) versus the installation of video surveillance cameras, pair composition, and the interaction between pair composition and the installation of cameras.

Rank	Model	df	logLik	AICc	ΔAIC_c_	AIC_w_	R^2^_m_	R^2^_c_
1	Age	5	− 466.963	944.087	0.000	0.560	0.872	0.877
2	Camera + age	6	− 466.306	944.837	0.751	0.385	0.878	0.882
3	Camera + age + camera * age	8	− 466.173	948.733	4.647	0.055	0.877	0.881
4	Camera	4	− 618.428	1244.938	300.851	0.000	0.015	0.074
5	Null model	3	− 620.136	1246.320	302.234	0.000	0.000	0.029

The productivity was modelled using a Poisson distribution and log link. The variables “territory” and “year” were included as random terms to account for non-independence of data.

*df* degrees of freedom, *logLik* log-likelihood, *AIC*_*c*_ Akaike Information Criterion for small samples, *ΔAIC*_*c*_ differences between AIC_c_ values of each model versus the best model, *AIC*_*w*_ Akaike weight, *R*^*2*^_*m*_ marginal R^2^, *R*^*2*^_*c*_ conditional R^2^.

Average breeding success was 66.80% ± 12.92 successful pairs all over the study period (2000–2022). Considering the period in which video surveillance cameras were installed (2021–2022), pairs under video surveillance showed lower average breeding success (mean = 33.33%, n = 12) than pairs without cameras (mean = 79.17%, n = 24).

## Discussion

Video surveillance cameras are a useful and practical tool to study many aspects of ecology and animal behaviour that would otherwise be practically impossible to obtain^1^. Today, thanks to technological advances, they constitute a cost-effective tool and their application and usefulness have been demonstrated over several decades of use, as reported in the literature^32,55^. Although most studies report neutral effects of cameras on birds^5,17,25,27^, or directly assume no effects without a formal test for it, very few studies analyse in detail the potential negative effects of their use, particularly on raptors. The advantages of using video cameras in nests are evident. However, as with all technologies, they have their limitations and drawbacks. Among them, it should be noted that the analysis of the images obtained through video surveillance is time-consuming since it requires long hours of viewing the recordings to obtain the data. In addition, the fact that they have to be installed outdoors inevitably exposes the technology to environmental conditions that are sometimes adverse (e.g., rain, wind, hail, humidity, salinity conditions or even the presence of other species that can alter the system such as herbivores and rodents) and for which the technology has not been originally designed, particularly in most cases where the equipment is readapted to operate outdoors. To this fact must be added the difficulty of camera installation, especially in the case of working at height such as on cliffs or trees where many species of birds nest (e.g. raptors). This entails the need to have to fine-tune all the parameters before installation, since any repair or readjustment that has to be done afterwards implies access to nests in the middle of the breeding season, coinciding with the most sensitive period for bird reproduction^56^. In this paper, thanks to the availability of a database obtained through long-term monitoring of a population of Bonelli’s eagle, I show how the inappropriate use of video surveillance cameras could result in negative effects on the reproduction of a threatened species. Far from being a critique of technology, this paper analyses the effect of video surveillance camera installation on reproduction and then discusses what factors might be behind the statistically significant reduction in breeding performance observed.

According to the results of this work, the installation of cameras in Bonelli’s eagle nests has caused a reduction in the productivity and breeding success of the territories under video surveillance. My results show no differences in breeding performance between the years 2021–2022, when cameras were operating, but significant differences in breeding parameters between territories under video surveillance and those without video surveillance in the same period. Considering the overall dataset (period: 2000–2022), there were no differences in breeding performance before and after the installation of video cameras. When all variables were taken into account, the results of the GLMM showed significant differences in productivity among territories in relation to the installation of video surveillance cameras and pair composition. Pairs composed of two subadult birds or by one subadult and one adult bird were less likely to be successful in reproduction, which is in agreement with the general pattern reported in this^39,57,58^ and other eagles^59,60^.

Interestingly, the installation of video surveillance cameras might affect the laying date. The two pairs that bred successfully of the four monitored in the first year showed an unusual delay in the laying date. Subsequently, the recovery on laying dates within the normal regime in the next breeding season might be attributed to the effect of habituation by the eagles^31^. Furthermore, in the case of the four pairs that were subjected to video surveillance in consecutive years, one of them changed the nest in the first breeding season to another cliff located 4.35 km from the usual breeding site; the second pair failed to breed in 2022 when it had two chicks in the nest, possibly due to a heavy rainstorm; the third pair, the one that unusually delayed reproduction in 2021, did not lay eggs in 2022; and the fourth pair did not lay eggs in either of the 2 years.

The potential causes of the observed reduction in breeding performance remain unknown. Based on the legal rights to access to environmental information in Spain, I requested the regional administration (Generalitat Valenciana) detailed information on the Bonelli’s eagle video surveillance project. Unfortunately, the technical specifications of the whole monitoring system (e.g., brand of the cameras, dimensions of the whole camera set, type of illumination) and, specifically, about the number of interventions done in each eagle’s territory once cameras were installed were not provided. Nevertheless, according to the specific information included in the forests rangers’ report and my field experience with the species all over the 23-years study period, I would take into account the following facts: (i) the installation of video cameras by forest rangers took place at the end of the year (Table 1), hence close to the laying date, coinciding with the mating phase that usually extends between 1 and 3 months before the laying date (from November to January in the study area) after the onset of the juvenile dispersal period of the chicks raised the previous year; (ii) the reiteration of visits to the nests once the cameras were installed to check the system (i.e., cables, cleaning of solar panels, unseating of the camera) or readjust the parameters (e.g., focus range of the camera), particularly during the incubation period and early stages of breeding (author’s personal observation); (iii) the installation of cameras in a particular area subject to constant human disturbance which results in larger home range and higher energy expenditure during some days of the week as reported by^42^; (iv) the installation of cameras in nests of unexperienced pairs composed by at least one subadult bird, with the consequent lower breeding experience^39,57,58^; and (v) a potential lack of experience in work at height of the team of forest rangers in charge of installing the cameras, which resulted in long operating times and the need to repeat new visits once the equipment was installed.

In some cases, breeding failure took place after the pair arranged the nest, even though no egg laying was observed. In other cases, video-surveilled pairs failed reproduction after incubation and egg hatching, when chicks were in the firsts week of development. Technical failures including the failure of the SIM card, power cable failure, lack of phone signal and even the deterioration of the antennas by herbivores made necessary reparation after camera installation. This implied repetitive visits the top of the cliff where the antennas and the transmission system were installed or even the nest. This reiteration of visits, along with the occurrence of natural events such as heavy rainstorm that affected the entire population during critical stages of the breeding season (i.e., when chicks were small with limited ability to termorregulation), in combination to the pair composition (i.e., some of the video-surveilled pairs were composed by at least one subadult bird, with the consequent lack of breeding experience) could potentially explain the decrease in breeding performance of video-surveilled pairs in comparison to non-surveilled pairs.

### Management recommendations

This paper is not intended to critique video camera technology for nest monitoring. On the contrary, the technology used by experienced researchers with clear scientific objectives constitutes an ideal complement to fieldwork. Therefore, this study is aimed at warning of the risks that the inadequate use of technology could have on endangered species if the necessary cautions are not taken into account. This includes the requirement of establishing clear scientific objectives, which were absent in this case. For example, the study of Bonelli’s eagle diet, which was the main justification for the installation of cameras in Bonelli’s eagle nests by the regional government, has been a topic deeply studied for decades (e.g.^12,61–72^, among many others). Therefore, the timeliness and novelty of the intervention, if other goals are not considered, could also be questioned.

In order to avoid unnecessary adverse effects, potential management actions to mitigate the effect of the installation of video cameras in nests on birds’ behaviour would include the need to plan the intervention dates in advance, separating them as much as possible from the mating period of the eagles. It would be also convenient to test the systems beforehand under controlled conditions or with non-threatened species in which a reduction in reproduction could not have significant effects on population dynamics. Ultimately, the post-installation monitoring is critical in order to avoid unnecessary disturbance to eagles and to ensure positive outcomes. In agreement with a similar study on the golden eagle (*Aquila chrysaetos*), cameras must be used judiciously because camera installation creates a persistent manipulation at the nest^13^. In addition, the use of video cameras should only be used as part of well-planned scientific studies, and researchers should follow protocols that minimize disturbance to eagles during installation and post-operational functioning.

Finally, I urge the scientific community to report the potential negative effects observed in their studies of the use of video surveillance cameras in bird nests, especially if the target species are threatened with extinction.

## Supplementary Information

Supplementary Figure S1.

## Data Availability

Due to the current endangered status of the species, data on nest locations are not publicly available. All data used in this study are available upon reasonable request to the author (Pascual López-López).
