# Supplementary material for: Potential negative effects of the installation of video surveillance cameras in raptors’ nests
Source: Sci Rep. 2022 Dec 20;12:21969. doi: 10.1038/s41598-022-26153-7 (PMC9768126; doi:10.1038/s41598-022-26153-7)
Supplement: Supplementary file 1 — Supplementary Figure S1. [file 41598_2022_26153_MOESM1_ESM.docx]

**Supplementary Materials**

**Potential negative effects of the installation of video surveillance cameras in raptors’ nests**

Pascual López-López^1*^

^1^Movement Ecology Lab, Cavanilles Institute of Biodiversity and Evolutionary Biology. University of Valencia. C/ Catedrático José Beltrán 2. E-46980. Paterna. Valencia. Spain.


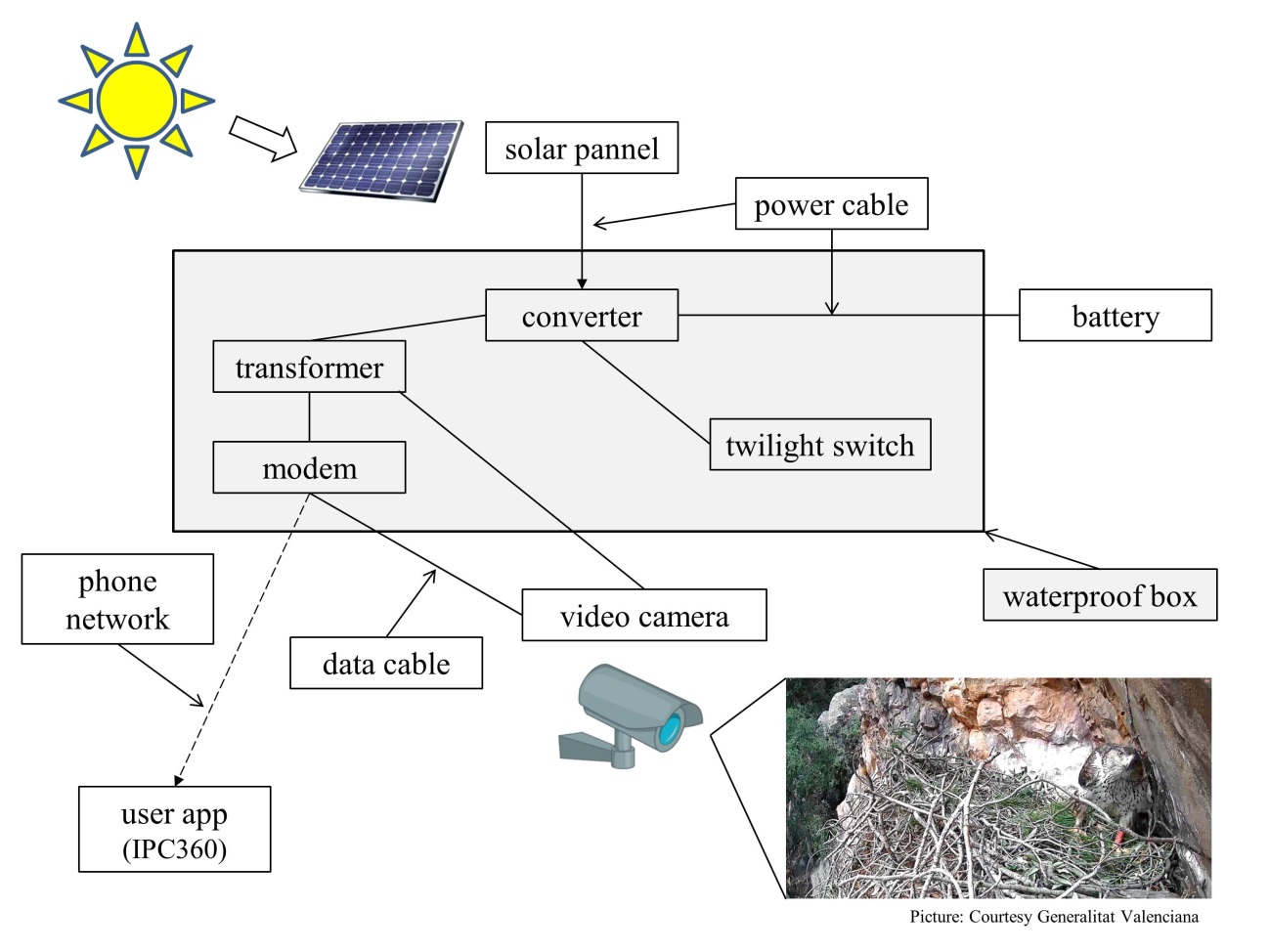


Figure S1.- Diagram of the video-monitoring system used to study Bonelli’s eagle (*Aquila fasciata*) in eastern Spain in 2021 and 2022.
